# Cryptic Population Structuring and the Role of the Isthmus of Tehuantepec as a Gene Flow Barrier in the Critically Endangered Central American River Turtle

**DOI:** 10.1371/journal.pone.0071668

**Published:** 2013-09-25

**Authors:** Gracia P. González-Porter, Jesús E. Maldonado, Oscar Flores-Villela, Richard C. Vogt, Axel Janke, Robert C. Fleischer, Frank Hailer

**Affiliations:** 1 Center for Conservation and Evolutionary Genetics, Smithsonian Conservation Biology Institute, National Zoological Park, Washington, District of Columbia, United States of America; 2 Museo de Zoología Facultad de Ciencias, Universidad Nacional Autónoma de México, México City, México; 3 Department of Vertebrate Zoology, National Museum of Natural History, Smithsonian Institution, Washington, District of Columbia, United States of America; 4 Instituto Nacional de Pesquisas da Amazônia, Coordinaçao de Biodiversidade, Coroado, Manaus, Brazil; 5 Goethe University Frankfurt, Institute for Ecology, Evolution and Diversity, Biologicum, Frankfurt am Main, Germany; 6 Biodiversity and Climate Research Centre and Senckenberg Gesellschaft für Naturforschung, Ecological Genomics, Frankfurt am Main, Germany; Tuscia University, Italy

## Abstract

The critically endangered Central American River Turtle (*Dermatemys mawii*) is the only remaining member of the Dermatemydidae family, yet little is known about its population structuring. In a previous study of mitochondrial (mt) DNA in the species, three main lineages were described. One lineage (*Central*) was dominant across most of the range, while two other lineages were restricted to Papaloapan (*PAP*; isolated by the Isthmus of Tehuantepec and the Sierra de Santa Marta) or the south-eastern part of the range (*1D*). Here we provide data from seven polymorphic microsatellite loci and the R35 intron to re-evaluate these findings using DNA from the nuclear genome. Based on a slightly expanded data set of a total of 253 samples from the same localities, we find that mtDNA and nuclear DNA markers yield a highly congruent picture of the evolutionary history and population structuring of *D. mawii*. While resolution provided by the R35 intron (sequenced for a subset of the samples) was very limited, the microsatellite data revealed pronounced population structuring. Within the Grijalva-Usumacinta drainage basin, however, many populations separated by more than 300 kilometers showed signals of high gene flow. Across the entire range, neither mitochondrial nor nuclear DNA show a significant isolation-by-distance pattern, but both genomes highlight that the *D. mawii* population in the Papaloapan basin is genetically distinctive. Further, both marker systems detect unique genomic signals in four individuals with mtDNA clade 1D sampled on the southeast edge of the Grijalva-Usumacinta basin. These individuals may represent a separate cryptic taxon that is likely impacted by recent admixture.

## Introduction

The Central American River Turtle, *Dermatemys mawii*, is the last surviving species of giant river turtles of the family Dermatemydidae. This turtle was an important economic and cultural resource for local people throughout its historical distributional range. Anthropogenic overexploitation by consumption of meat has caused drastic population declines [1], [2], [3], and the species has been listed as critically endangered since 2005 by the IUCN [4] and is also listed in the US Endangered Species Act [4], [5], and on Appendix II of CITES [4], [6].

**Figure 1:**
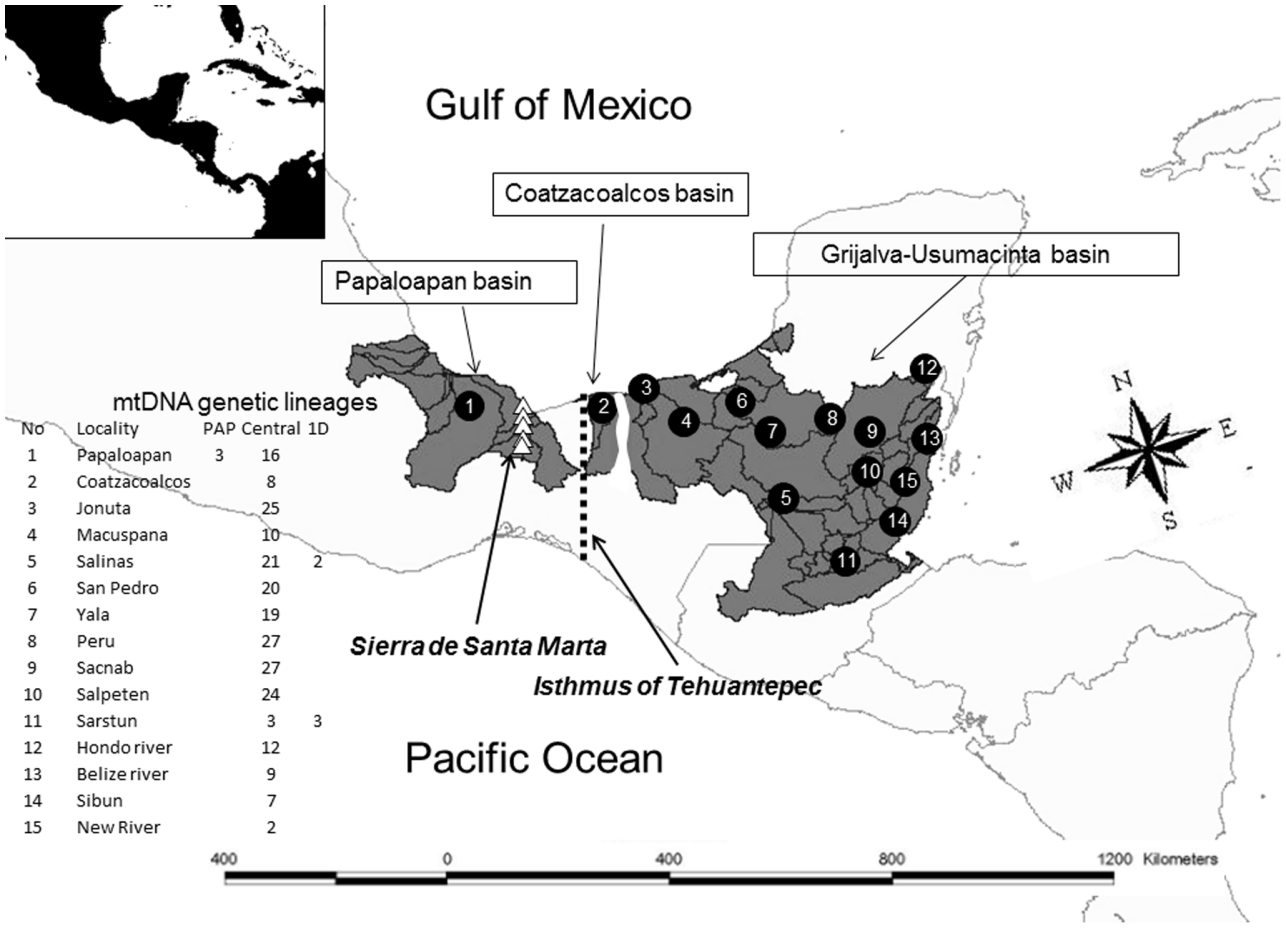
Geographic distribution of *D. mawii* (dark gray shading). The main river basins, distribution nuclei, and biogeographic barriers in the region are shown. Numbers 1 to 15 denote the sampling localities. PAP, “Central” and 1D are the three mtDNA lineages [13]. Dark lines correspond to rivers.

The distribution range of *D. mawii* includes parts of Belize, the Atlantic Coast of Guatemala and large parts of the Mexican States of Veracruz, Tabasco, Campeche, Chiapas and the southern part of Quintana Roo (Fig. 1). *Dermatemys* occurs in three of the largest watersheds of Mesoamerica, the Papaloapan, the Coatzacoalcos and the Grijalva-Usumacinta river basins [7], [8]. Notably, this area spans the Isthmus of Tehuantepec and the Sierra de Santa Marta which are known to be major biogeographic breaks in Mexico [9] for a variety of animal taxa [10], [11], [12], [13]. For example, in the genus *Bufo* there are different genetic lineages at the west and east of the Isthmus of Tehuantepec and Sierra de Santa Marta [12]. Even species occurring in apparently homogeneous lowland habitats in Mesoamerica can exhibit population genetic structure when movement is inhibited. The red-eyed tree frog (*Agalychnis callidryas*) of lower Mesoamerica shows pronounced genetic structure not only across the Cordilleran Mountains, but also within each of the contiguous Caribbean and Pacific coastal forest habitats [14]. *Dermatemys mawii* might therefore show previously unknown genetic structuring across its range, with potential conservation implications.

This study is part of a long-term conservation project to develop management plans by identifying management units (MUs [15]) for ensuring the long-term maintenance of *D. mawii*. Recently, a study that characterized mtDNA variation in the species found signatures of phylogeographic structuring and phylogenetic splits among lineages up to a divergence of 2% [16]. A divergence of 2% at mtDNA loci in turtles can warrant species-level distinction [17]. Most mtDNA haplotypes belonged to a diverse central lineage (“Central”), but two strongly divergent lineages were found in the Northwest (Papaloapan drainage basin, “PAP”) and the southeastern edge of the Grijalva-Usumacinta basin (“1D”) respectively. It was hypothesized that this ancient structuring has been secondarily blurred by extensive gene flow. Notably, this secondary homogenization of genetic structuring also occurred across two major hydrological divides that cause biogeographic breaks in other aquatic Mesomerican taxa [12], [13]. Two populations (Sarstun and Salinas) were found to harbor *D. mawii* individuals (total: n = 4) carrying a rare, divergent mtDNA haplotype (1D), co-occurring with individuals that carry common haplotypes of the “Central” clade. Based on single-locus evidence from maternally inherited mtDNA, however, it was not possible to assess whether members of the different mitochondrial genetic lineages are capable of interbreeding with each other. Microsatellite markers are commonly used to assess population genetic structuring, impacts of demographic bottlenecks [18], [19], [20], effective population sizes and migration rates [20], and to identify MUs [21].

Nuclear DNA markers have been used to complement the results of mtDNA studies with a bi-parental perspective in other population genetic studies of turtles. For instance, *Emys orbicularis* shows low divergence and lack of population genetic structure throughout the Iberian Peninsula when analyzed using mtDNA, while results of nuclear microsatellite loci revealed relatively high levels of genetic variability within populations and population structuring [22]. In the Arrau turtle (*Podocnemis expansa*), the overall differentiation among populations estimated by *F*_ST_ values was fourfold greater for mtDNA than for nuclear microsatellite markers [23]. Because mitochondrial genes are maternally inherited as a single linkage group (haplotype), they reflect only some aspects of a species' evolutionary history [24]. In contrast, a set of independently inherited nuclear microsatellite loci can provide an independent view [25]. It is thus important to add nuclear markers to an analysis of mtDNA, when assessing levels of connectivity and evolutionary distinctiveness among populations.

In this study we investigate genetic variation at 7 polymorphic microsatellite loci and one nuclear intron in *D. mawii* populations from across the species' distribution range. We test to what extent the results from the nuclear genome show concordance with previously published mtDNA structuring [16]. Assessing and maintaining genetic diversity is pivotal for conservation management plans that aim to protect the evolutionary potential of the species. Understanding the distribution and variation of that diversity at various spatial scales (basin, river and populations) improves the efficiency of conservation and recovery efforts. Therefore, a main goal of this study was to provide information for the conservation and management of the Central American River Turtle by achieving the following objectives: (1) to assess genetic diversity within populations of this endangered species using polymorphic microsatellite loci, (2) to analyze gene flow and genetic structure among populations, defining MUs for the optimal genetic management of *D. mawii*, (3) to determine if *D. mawii* populations have genetic signatures of a recent bottleneck(s), and (4) to compare the patterns observed at microsatellite loci with those recovered previously from mtDNA [16].

## Materials and Methods

### Field and laboratory methods

We collected 253 tissue samples from the hind-foot webbing by cutting small skin snips (3 mm^2^). Before cutting, the area was disinfected with 62% ethanol gel and, after sampling, we covered the wound with antiseptic “new skin” (8-hydroxyquinoline at 1%) in order to avoid infections. We avoided cross-individual contamination by using scissors thoroughly cleaned with a 10% bleach solution. We followed the guidelines for use of live amphibians and reptiles in field research from the American Society of Ichthyologists and Herpetologists (ASIH), the Herpetologists' League (HL) and the Society for the Study of Amphibians and Reptiles (SSAR) [26]. All animal work was conducted according to the guidelines from the Committee on Ethics of Animal Experiments at the Philadelphia Zoo and the USFWS for approval of the methods for capturing and handling the animals (Permit Numbers: #MA095827-0; MA119634-0/06; MA139456-0/07; MA194812-0/08; EN/FIS/15/04/08(10)Vol1). All analyzed samples came from adult individuals, and all animals were released at the point of collection. Immediately after collection, tissues were preserved in 70% ethanol and later stored in a freezer at −80°C. Sampling took place between 2004 and 2009 and covered most of the current geographic range of this species (Fig. 1). Samples were collected from 15 localities within the following major river drainages (Fig. 1): the i) Papaloapan and ii) Coatzacoalcos river basins, and iii) the Salinas and Hondo rivers and the Grijalva and Usumacinta basin in Mexico; Laguna Peru, Laguna Sacnab, Laguna Salpeten, Laguna Yala, the San Pedro River and the Sarstun River in Guatemala; and from the Belize River, New River Lagoon, and Sibun River in Belize. These samples include all individuals previously analyzed for mtDNA, plus 15 additional individuals that did not amplify for mitochondrial loci [16]. We isolated DNA from tissue samples with DNeasy extraction kits (QIAGEN) and proteinase K.

We amplified seven dinucleotide-repeat polymorphic microsatellite loci that we previously designed for *D. mawii* (*Dm3A-32, Dm3A-37, Dm3A-58, Dm3A-13, Dm3A-17, Dm3A-72, Dm3A-42*) [27], and an eighth, *GP96*, that was designed for *Gopherus polyphemus* but cross-amplifies in other turtle species [28]. The forward primers were fluorescently labeled (HEX or FAM). DNA was amplified in singleplex PCR reactions using the following conditions: for *Dm3A-32*, *Dm3A-37*, *Dm3A-58*, *Dm3A-13*, *Dm3A-17*, and *Dm3A-42*, an initial step at 94°C for 7 min was followed by 45 cycles of 92°C for 1 min, annealing at 50–65°C for 1 min depending on the specific primer pair (50°C for *Dm3A-58* and *Dm3A-13*; 55°C for *Dm3A-32*, *Dm3A-37* and *Dm3A-42*), extension at 72°C for 1 min, and a final extension at 72°C for 7 min. The conditions for the locus *Dm3A-72* were: one initial step at 94°C for 7 min, followed by 38 cycles of 94°C for 40 seconds, annealing at 64°C for 40 seconds, 72°C for 45 seconds, and a final extension at 72°C for 7 min. The conditions for locus *GP-96* were: 95°C for 7 min, followed by 38 cycles of 95°C for 40 s, 50°C for 30 sec, 72°C for 1 min, and a final extension at 72°C for 15 min. The resulting PCR products were analyzed on a 3130XL Genetic Analyzer using GeneScan ROX 500™ size standard and genotyped with GeneMapper 4 software (Applied Biosystems).

We also sequenced 779****bp of the first nuclear intron of the RNA fingerprint protein R35 in 11 samples from the three main basins, covering the three main mtDNA lineages (6 individuals from the Central lineage, 1 from PAP and 4 from 1D), comparing our data to a sequence previously used in a phylogenetic study of turtles [29] (Genbank entry AY339638). The R35 intron has previously been shown to be a single-copy locus that provides excellent resolution among turtle species, although it has a slower mutation rate than mitochondrial DNA markers [26]. This was done in particular to further elucidate the evolutionary significance of the highly divergent mtDNA haplotype 1D [16]. PCR conditions were as in [29]: 94°C for 5 min, followed by 35 cycles of 94°C for 30 sec, 60°C for 90 sec, 72°C for 2 min, and a final extension at 72°C for 10 min. Products were cleaned using ExoSAP-IT® (USB Scientific), cycle sequenced using BigDye® terminator v3.1 (Applied Biosystems), cleaned with Sephadex ® (Sigma-Aldrich) and run on a ABI3130XLGenetic Analyzer (Applied Biosystems).

### Statistical analyses

#### Genetic diversity within localities

We calculated the mean number of alleles (MNA) per locus, allele frequencies, expected (H_E_) and observed (H_O_) heterozygosities [30], and tested for deviations from Hardy-Weinberg (HW) expectations globally for each population and separately for each locus by performing the exact test of Guo and Thompson [31], using GENEPOP on the web version 4.0.10 [32]. Sequential Bonferroni correction [33], [34] was used to reduce the number of type I errors (false positives) for multiple comparisons (α = 0.05). We also used exact tests in GENEPOP to investigate signals of linkage disequilibrium (LD) among loci, again applying sequential Bonferroni correction. We checked for null alleles using MICROCHECKER v 2.23 [35]. Locus *Dm3A-17* was excluded from the study because of a significantly lower than expected observed heterozygosity in most populations, indicating the widespread presence of null alleles at this locus. Our analyses are therefore based on the 7 loci that did not deviate from neutral HW expectations.

We used the program HP-RARE [36] to assess allelic richness (A_R_) within localities. HP-RARE uses rarefaction approach to account for differences in sample size among populations. As a baseline, we standardized sample size to the smallest number of samples per locality included in this analysis (Sibun, with 7 diploid individuals, i.e. 14 alleles used as baseline sample size) and then determined, using rarefaction, the expected number of alleles at each locality. We also used HP-RARE to determine the private allelic richness (PA [37]). Due to the low sample sizes for Sarstun (n = 5) and New River (n = 2), these populations were discarded for rarefaction analysis in HP-RARE. In addition, New River was disregarded in the isolation-by-distance correlations and assignment tests.

#### Gene flow and genetic structure

Levels of nuclear DNA differentiation among populations were calculated based on estimates of the fixation index Θ [38] in the program GENETIX 4.05.2 [39]. Assignment tests of individual turtles to specific populations were conducted with GENECLASS 2.0 [40], [41]. The employed procedure uses a Bayesian approach that computes the composite likelihood of an individual multi-locus genotype belonging to a candidate set of populations, assigning each individual to the population where the likelihood of its genotype is highest. Isolation by distance was tested by regression of the genetic distance (F_ST_/(1-F_ST_) [42] between pairs of populations against the logarithm of the geographic distance, measured in kilometers, between them. The statistical significance of this correlation was determined by Mantel tests in the Isolation By Distance Web Service IBDWS v 3.16 software [43].

We used the program STRUCTURE v2.3.1 [44] to determine population genetic structuring without pre-assigning individuals to sampling localities. This model-based (Bayesian) approach performs individual clustering for K distinct clusters, given a prior distribution of the allele frequencies in each population. We explored scenarios with K = 1 to 12 populations using 250,000 iterations as burn-in followed by 500,000 iterations for parameter estimation, based on the admixture model and assuming correlated allele frequencies. Each run was performed 8 times, and results were processed with STRUCTURE HARVESTER v0.56.4 [45], which also calculated the ΔK of Evanno et al [46]. Results of different replicates were combined and averaged using CLUMPP v1.1.2 [47].

Haplotypic phase of the sequences from the nuclear R35 intron was obtained from the program PHASE, as implemented in DNASP 5.01 [48]. The software IMGC [49] was used to determine the longest recombination-free sections of nuclear DNA. A statistical parsimony network of R35 intron haplotypes was obtained using TCS 1.21 [50], [51], using the 95% credibility limit.

#### Demographic tests

Evidence of recent population reductions was assessed using BOTTLENECK v 1.2.02 [52]. This program computes the distribution of the heterozygosity expected from the observed number of alleles (k), given the sample size (n) under the assumption of mutation-drift equilibrium for each population sample and for each locus. Population bottlenecks induce a temporary excess of heterozygosity compared to the expected equilibrium value. This distribution was obtained through simulating the coalescent process of n genes under two mutation models, the two phase model (TPM; [53]), and the stepwise mutation model (SMM; [54]). Significance was assessed from two-tailed Wilcoxon signed-rank tests (α = 0.05) [55]. We also tested for mode-shift away from an L-shape distribution of allelic frequencies [56]. In both of these tests, we disregarded all localities with less than 15 sampled individuals, because bottleneck tests are sensitive to sample size.

#### Correlation analysis of mtDNA and nuclear DNA

In order to analyze the possible concordance between the results of F_ST_ values estimated by Gonzalez-Porter et al. [16] with mtDNA (Cyt*b* and ND4 markers), against those estimated using microsatellites, we conducted a correlation analysis using Mantel tests. A Factorial Correspondence Analysis (FCA) was conducted in GENETIX 4.05.2 [36], based on the microsatellite multi-locus genotypes. To graphically visualize the differentiation between populations, we used the arrangement of the three lineages based on mtDNA found by Gonzalez-Porter et al. [16]: PAP with three haplotypes (4E, 5E, 7E), haplotype 1D, and a Central lineage containing the rest of haplotypes (2A, 2I, 3A, 5A, 5B, 5C, 5F, 5G, 5H, 5J, 6A, and 7A).

## Results

### Genetic diversity within localities

A total of 253 individuals from 15 localities were genotyped at 7 polymorphic microsatellite loci (another microsatellite, *Dm3A-17*, was excluded due to strong deviations from HW expectations; see above). Among the 7 remaining microsatellites, no locus alone deviated significantly from HW equilibrium frequencies (p>0.05 after sequential Bonferroni correction) at any locality. Across loci, and following sequential Bonferroni correction, only the 25 Salinas samples deviated significantly from HW equilibrium (P = 0.003) (Table 1). For this population, two loci (*Dm3A32* and *Dm3A13*) showed significant heterozygote deficit (p<0.05; but non-significant after sequential Bonferroni correction). This population harbors genetic material from an apparently differentiated lineage (mtDNA 1D individuals which appear as outliers in FCA tests; see below), which may explain these deviations from HWE. We did not find any evidence of significant linkage disequilibrium between any pair of loci at any locality (p>0.05, applying sequential Bonferroni correction). No locus exhibited large-allelic dropout as assessed in MICROCHECKER. We thus kept all remaining 7 loci for further analyses. These 7 microsatellite loci exhibited a total of 72 alleles, with an allelic richness (A_R_) ranging from 2.1 at Salpeten to 4.5 for Jonuta. Private allelic richness (PA) ranged from zero in Peru and Yala, to 0.64 in Papaloapan. Observed heterozygosity ranged from 0.329 for Sacnab to 0.609 for Jonuta (Table 1). The latter appeared to be the most diverse population with the highest levels of allelic diversity and heterozygosity, while Sacnab was the least diverse population for both of these measures.

**Table 1:** Genetic diversity within populations, grouped by river basin.

River basin	Locality	N	H_E_	H_O_	A_R_ (14)	PA (14)
**Papaloapan**	**Papaloapan**	23	0.522	0.497	3.8	0.64
**Coatzacoalcos**	**Coatzacoalcos**	8	0.455	0.482	3.7	0.22
**Grijalva- Usumacinta**	**Jonuta**	27	0.570	0.609	4.5	0.30
**“”**	**Macuspana**	11	0.531	0.507	4.2	0.12
**“”**	**Salinas**	25	0.522	0.463*	4.3	0.29
**“”**	**San Pedro**	21	0.375	0.415	3.1	0.03
**“”**	**Yala**	20	0.437	0.414	3.3	<0.01
**“”**	**Peru**	28	0.399	0.434	3.0	0.12
**“”**	**Sacnab**	30	0.317	0.329	2.4	0.03
**“”**	**Salpeten**	25	0.309	0.343	2.1	<0.01
**“”**	**Sarstun**	5	0.491	0.429	n/a#	n/a#
**“”**	**Hondo River**	12	0.457	0.571	2.9	0.09
**“”**	**Belize River**	9	0.531	0.556	3.3	0.03
**“”**	**Sibun**	7	0.436	0.490	2.9	0.14
**“”**	**New River**	2	0.304	0.357	n/a#	n/a#

N sample size, H_E_ and H_O_ average (multilocus) expected and observed heterozygosity, A_R_ rarefied allelic richness, PA rarefied private allelic richness (both based on a subsample of 14 alleles, corresponding to 7 diploid individuals).

* p<0.05; significant heterozygote deficit.

# values not reported due to small sample size.

### Gene flow and genetic structure

Overall genetic differentiation at microsatellite loci was moderate, with a global Θ_ST_ [38] of 0.118 (95% confidence intervals from bootstrapping by loci: 0.070–0.164). Most pairwise comparisons among populations (localities) were significant (p≤0.05; Table 2), with only 9 out of 105 values being non-significant. The samples from Papaloapan, the only population in our study situated west of the Isthmus of Tehuantepec (Fig. 1), showed high levels of differentiation compared to almost all other populations (values ranged from 0.1 to 0.3), and also had the largest number of private allelic richness (0.64; Table 1). Also the samples from Sibun, Salpeten and Sacnab, all located near the eastern edge of the distribution, showed relatively high differentiation from the remaining populations. For Sibun, this might potentially be related to the small sample size (n = 7), but the remaining two were based on samples size of 25 and 30, respectively, and still showed reduced genetic diversity (Table 1). All low and/or non-significant differentiation values were found in pairwise comparisons among sites in the Grijalva-Usumacinta basin, even across distances larger than 300 kilometers (Fig. 1; Table 2). We found no correlation between genetic and geographic distance across our data set (Fig. S1), as indicated by a Mantel test (Z = 1.56, r = 0.007, one sided p = 0.515).

**Table 2:** Matrix of pairwise genetic differentiation among localities (Weir & Cockerham's [38] Θ_ ST_).

	Pap	Coa	Jon	Mac	Sali	SP	Yal	Per	Sac	Salp	Sar	Hon	Bel	Sib	NR
**Pap**	-	**0.133**	**0.122**	**0.152**	**0.119**	**0.228**	**0.195**	**0.205**	**0.289**	**0.300**	0.103	**0.140**	**0.196**	**0.253**	**0.206**
**Coa**		-	0.025	0.050	0.034	0.084	0.067	0.093	**0.173**	**0.153**	0.090	0.065	0.077	**0.229**	**0.164**
**Jon**			-	0.018	0.016	0.066	0.054	0.075	**0.148**	**0.162**	0.082	0.054	0.070	**0.193**	**0.135**
**Mac**				-	*0.020* *	0.077	0.057	0.069	**0.181**	**0.188**	0.059	0.069	0.105	**0.207**	**0.121**
**Sali**					-	0.061	0.051	0.066	0.134	**0.169**	*0.020* *	0.037	0.091	**0.222**	**0.133**
**SP**						-	*−0.008* *	*0.006* *	0.061	0.073	**0.149**	0.053	0.064	**0.280**	**0.187**
**Yal**							-	*0.005* *	0.078	0.089	0.094	*0.023* *	0.037	**0.200**	0.093
**Per**								-	0.104	0.102	**0.126**	0.068	0.065	**0.253**	**0.173**
**Sac**									-	**0.142**	**0.195**	0.075	0.112	**0.382**	**0.322**
**Salp**										-	**0.257**	**0.118**	0.102	**0.281**	**0.192**
**Sar**											-	*0.004* *	**0.135**	**0.187**	0.067
**Hon**												-	0.043	**0.149**	*0.027* *
**Bel**													-	**0.154**	**0.166**
**Sib**														-	*0.035* *
**NR**															-

Values higher than the average level of differentiation (across all populations: Θ_ST_ = 0.118) are shown in bold and underlined.

* = Non significant (p>0.05), assessed from 1000 randomizations (values shown in italics).

Pap = Papaloapan, Coa = Coatzacoalcos, Jon = Jonuta, Mac = Macuspana, Sali = Salinas, SP = San pedro, Yal = Yala, Per = Peru, Sac = Sacnab, Salp = Salpeten, Sar = Sarstun, Hon = Hondo River, Bel = Belize River, Sib = Sibun, and NR = New River.

Standard assignment test results revealed that 155 of 251 (61.8%) of individuals were assigned to their known (original) collection localities, and most cases of mis-assignment were to neighboring populations from within the same river basin (Table 3). The sites with the highest percentages of correctly assigned individuals were Sibun with 100% (n = 7) and Papaloapan with 91% (n = 23), likely reflecting the above noted differentiation of these populations, and Sacnab with 86% (n = 30; note its limited variability; Table 1). The sites with the lowest percentages of correctly assigned individuals were Yala with 25%, San Pedro with 29%, and Belize River with 44%, three neighboring populations from the Yucatan peninsula.

**Table 3:** Results of the assignment test.

Locality*	Pap	Coa	Jon	Mac	Sali	SP	Yal	Per	Sac	Salp	Sar	Hon	Bel	Sib	N	% self
**Pap**	**21**	2	-	-	-	-	-	-	-	-	-	-	-	-	23	91.3
**Coa**	-	**6**	2	-	-	-	-	-	-	-	-	-	-	-	8	75.0
**Jon**	1	-	**16**	3	-	2	-	1	1	-	-	-	-	1	27	59.3
**Mac**	-	-	1	**6**	-	1	1	2	-	-	-	-	-	-	11	54.6
**Sali**	1	-	-	2	**14**	2	-	-	-	-	3	2	-	1	25	56.0
**SP**	-	-	1	-	1	**6**	2	5	2	2	-	1	1	-	21	28.6
**Yal**	-	-	1	-	-	6	**5**	3	2	-	-	-	2	1	20	25.0
**Per**	-	-	-	-	2	5	2	**13**	2	1	-	1	1	1	28	46.4
**Sac**	-	-	-	-	-	1	2	-	**26**	-	-	1	-	-	30	86.7
**Salp**	-	-	-	-	-	3	-	1	1	**18**	-	-	1	1	25	72.0
**Sar**	-	-	-	-	-	-	-	-	-	-	**4**	-	-	1	5	80.0
**Hon**	-	-	-	-	-	-	2	-	1	-	-	**9**	-	-	12	75.0
**Bel**	-	-	-	-	1	-	-	-	1	-	1	2	**4**	-	9	44.4
**Sib**	-	-	-	-	-	-	-	-	-	-	-	-	-	**7**	7	100.0

**Sample origins (sampling localities of each sample) are shown in rows, and estimated source populations in columns. N is the number of samples from each locality, %self is the self-assignment rate (% of samples assigned to the known sampling locality).**

* Pap – Papaloapan, Coa – Coatzacoalcos, Jon – Jonuta, Mac – Macuspana, Sali – Salinas, SP – San Pedro, Yal – Yala, Per – Peru, Sac – Sacnab, Salp – Salpeten, Sar – Sarstun, Hon – Hondo, Bel – Belize, Sib – Sibun.

**Figure 2:**
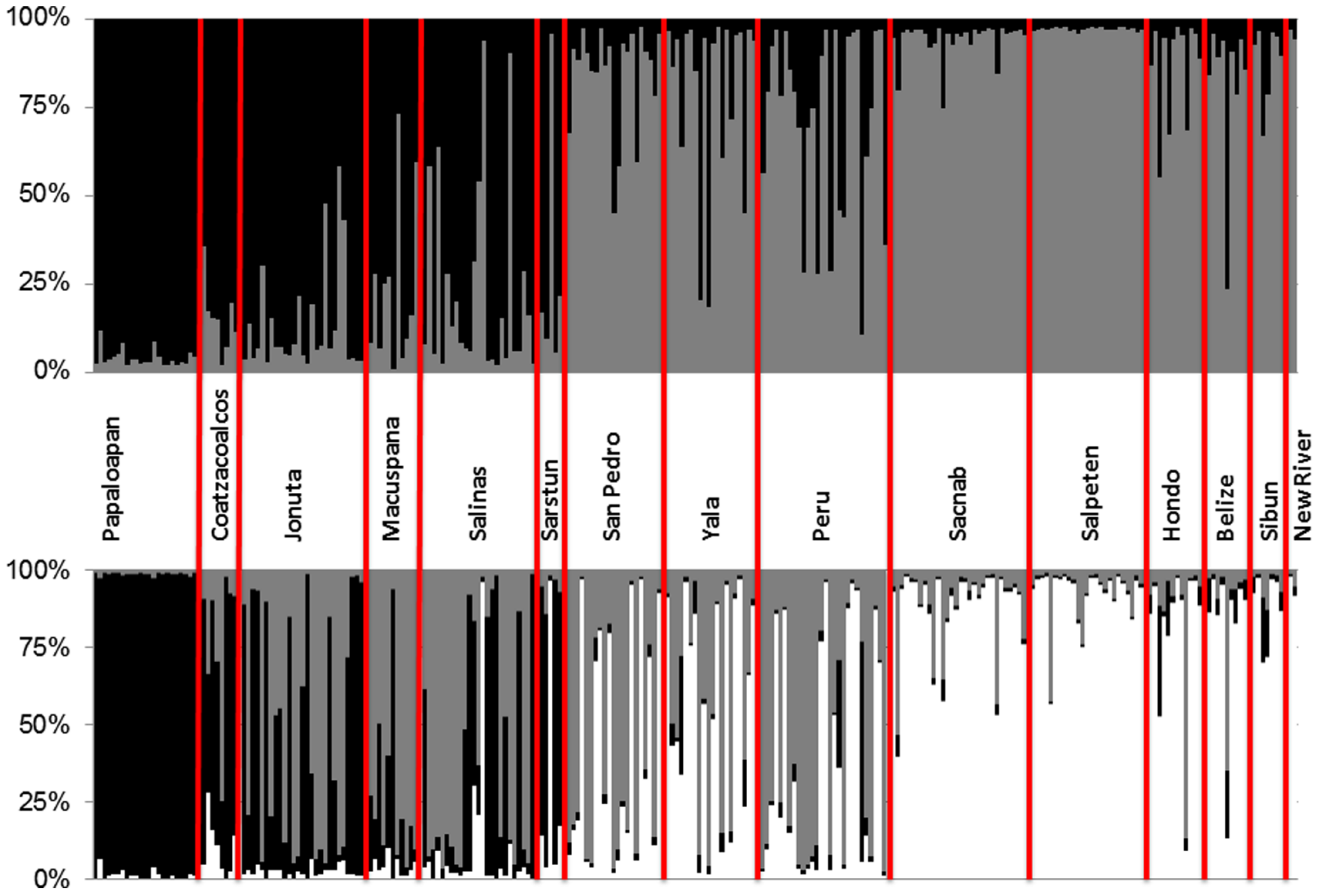
Individual clustering results from STRUCTURE for K = 2 (above) and K = 3 (below) clusters. Data from individuals is shown in columns, with shading corresponding to cluster membership (in %).

**Figure 3:**
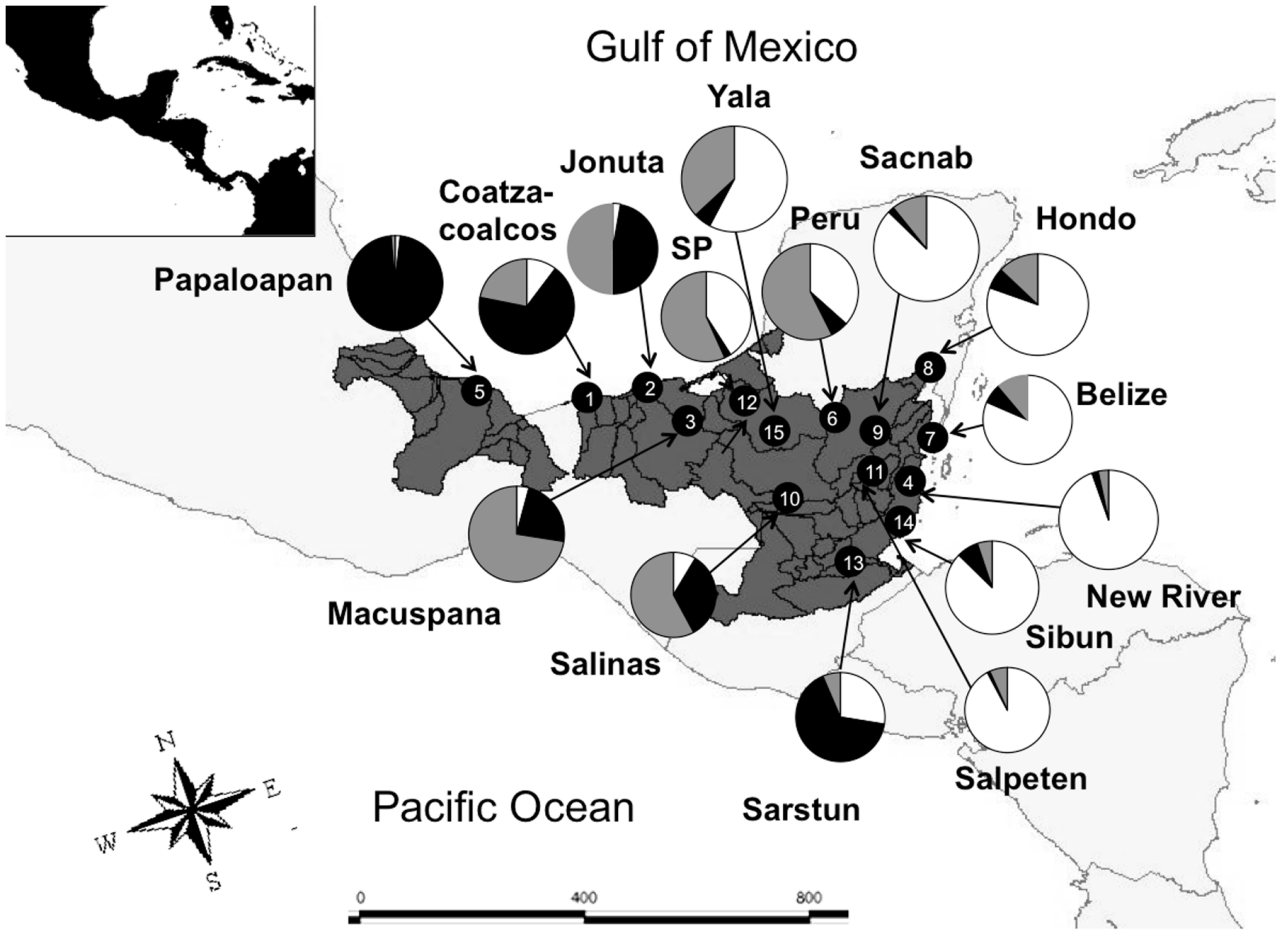
Results from STRUCTURE for K = 3, plotted by locality on a map. One cluster (black) prevails in Papaloapan and other adjacent populations in the (south-)west, one cluster (gray) is most common in centrally located populations, and a third cluster (white) is dominant in the region of the Yucatan peninsula.

ΔK, the statistic of Evanno et al. [46], was highest at K = 2 (see Fig. S2), favoring the STRUCTURE results for 2 clusters. At this clustering level, most populations appeared to be admixed, containing mixed genotypes from both clusters (Fig. 2). The two clusters showed a (south-)west to (north-)east gradient, roughly separating the Yucatan populations from the others (Fig. 2). At K = 3 (where lnP(D) was higher than for K = 2; Fig. S3) another cluster appeared, with individual cluster memberships in Papaloapan nearing 100%, and only a few individuals – also from the (south-)western part of the range – showing high membership in this cluster (Fig. 2; Fig. 3). The Yucatan cluster from K = 2 was also visible at K = 3 (Fig. 2), and a third cluster was most prominent in some populations from the west and north (except Papaloapan).

### Population reduction test

No signal of a recent bottleneck was detected in any of the reasonably extensively sampled localities (n≥15) using BOTTLENECK: no comparison was significant for the Wilcoxon test or the Mode-shift test for the TPM or SMM models.

### Correlation between mtDNA and nuclear DNA

Across all localities, patterns of nuclear genetic differentiation were concordant with results obtained using mtDNA from the same 15 populations [16]. Pairwise nuclear F_ST_ values were significantly correlated with pairwise mtDNA Φ_ST,mt_ values from the same localities (based on a Mantel test, evaluating significance from 20,000 randomizations: Z = −12.63, r = −0.23, p = 0.038).

**Figure 4:**
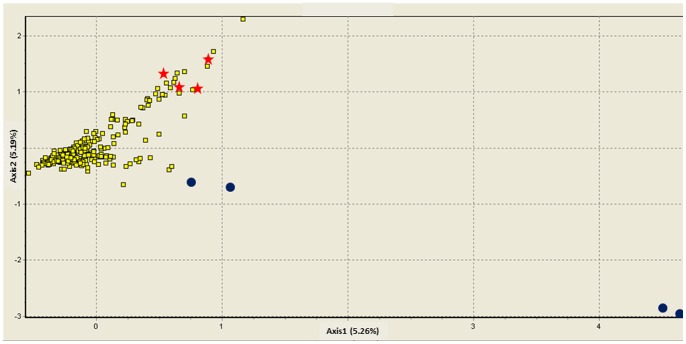
Factorial correspondence analysis (FCA). Results for all individuals based on multilocus microsatellite genotypes. Individuals are coded according to their mtDNA lineages; PAP (red stars), 1D (blue dots), and “Central” (yellow squares). The axes 1 and 2 each explain just over 5% of the total variation in the data set. The two outliers on the right are both homozygous for rare alleles (179/179 at locus *Dm3A32*, and 250/250 at *Dm3A13*).

The results from mtDNA in *D. mawii* [16] revealed the presence of three divergent mitochondrial lineages, one (1D) found in Salinas and Sarstun, a second lineage (“Papaloapan”: haplotypes 4E, 5E and 7E) restricted to the Papaloapan basin, to the West of the Sierra de Santa Marta (Fig. 1), and a third widespread lineage (“Central”), that was found in all studied localities. Compared to other pairwise comparisons (Table 2), the Salinas and Sarstun populations (where mtDNA haplotype 1D was found) did not appear to be especially strongly differentiated from other populations (where the 1D haplotype was absent) at the surveyed microsatellite loci, when averaging across all individuals from those populations. However, an individual-based FCA revealed that all four individuals which carried the mitochondrial 1D haplotype appeared distinct or very distinct (Fig. 4): two individuals from Salinas were strongly differentiated from all others, while the two other 1D-individuals from Sarstun were placed outside the variability of all other individuals, but less strongly separated from them than the first two. Based upon this analysis, we conclude that in general, individuals with the mitochondrial haplotype 1D are highly divergent from the rest of the individuals. However, the individuals carrying “Papaloapan” type mtDNA haplotypes did not appear differentiated from the “Central” type individuals (Fig. 4).

Sequences from the nuclear R35 intron showed no signal of recombination in the IMGC test, so we used the entire 779****bp fragment in PHASE to determine haplotypes. Sequences obtained for *D. mawii* have been deposited in Genbank (accession numbers JN655665-JN655668). Only three sites were variable in the alignment, limiting the power to detect any population differentiation. We found four haplotypes (Table 4), three in our samples, and a fourth that corresponded to the sequence downloaded from GenBank. A statistical parsimony network from TCS showed that the four haplotypes were related in a star-shape manner, with a central haplotype (haplotype 2) being separated by one mutational step from each of the remaining haplotypes. The three haplotypes we found in our samples did not reveal any clear phylogeographic structure, with the most common haplotype (haplotype 2) present in individuals from populations all across the distribution range. The four individuals with mtDNA haplotype 1D all carried the R35 haplotype 2, in common with individuals from the “Central” mtDNA lineage.

**Table 4:** Haplotypes of the *R35* intron [29] sequenced in a subset of individuals.

R35 haplotype	individuals	haplotypes mtDNA	mtDNA clades	Localities
**1**	Phi1, Phi4, 52, 518	1D	1D	Sarstun, Salinas
	13*, 118*	3A, 7A	Central	Salinas
**2**	13*, 80*, 87, 118*, 325	3A, 5A, 6A, 7A, 4E	Central, PAP	Salinas, Jonuta, Papaloapan
**3**	1, 80*	2A, 5A	Central	Salinas, Jonuta
**4**	GenBank AY339638	1D	1D	Sarstun (via Philadelphia zoo)

A summary of mtDNA data for the same individuals [16] is given for comparison.

* heterozygous individuals.

## Discussion

In this study we used microsatellite loci to examine genetic structure of the Mesomerican endemic *D. mawii* at a finer scale than was possible using only mtDNA data from the same 15 populations [16]. With this higher resolution from independently inherited nuclear loci we were able to detect pronounced genetic differentiation among some populations, even within the same river basin.

Differentiation patterns among populations were significantly correlated between mtDNA and microsatellites, suggesting that the markers picked up corresponding evolutionary signals. Also, both marker systems provided evidence of distinctive alleles in individuals from Sarstun/Salinas, while failing to detect significant range wide patterns of isolation by distance. Differentiation at both microsatellite and mtDNA markers revealed the historically isolating effects of the Isthmus of Tehuantepec and the Sierra de Santa Marta on *D. mawii*, but current population structuring suggests some gene flow across this barrier. Although shared ancestral alleles could lead to similar patterns, some level of previously existing population structuring in *D. mawii* may have been secondarily blurred by wide-ranging, and possibly human-mediated, gene flow. High levels of gene flow were also detected across much of the Yucatan peninsula, and among some populations that are separated by long distances across the Grijalva-Usumacinta basin.

These results are similar to those found in another turtle species, *Podocnemis expansa*, which lacks association between genetic and geographic distance when using mtDNA and microsatellites [23]. This species also inhabits Neotropical rivers and is capable of moving great distances, similar to *D. mawii*.

### Variability within populations: demographic effects on long-living organisms

Genetic variability did not vary drastically among populations, although some populations in the eastern parts of the distribution appeared to have somewhat reduced levels of variation (e.g., Sacnab and Salpeten; Table 1). These populations also showed relatively strong genetic differentiation from other *D. mawii* populations (Table 2). Given the low frequency of private alleles in Sacnab and Salpeten, recent drift and/or demographic isolation likely explain these signals. It is noteworthy that recent census data from *D. mawii* have documented that anthropogenic overexploitation (consumption of meat) has caused drastic population declines [1], [2], [3].

However, the population reduction tests indicated that none of the analyzed *D. mawii* populations showed significant evidence of recent demographic bottlenecks. The long generation time and delayed sexual maturity of *D. mawii* (mean lifespan is greater than 50 years) have likely buffered against rapid loss of genetic variability during phases of population size reduction [57], [58]. The same phenomenon is evident in *Podocnemis unifilis* populations in the Orinoco and Amazon basins [59]. As in numerous other species with a long generation time, significant levels of genetic diversity may thus be retained through demographic fluctuations, allowing for development of conservation actions as long as demographic extinction risk is not too high [18], [57].

### Isolating effects of the Isthmus of Tehuantepec and the Sierra de Santa Marta

Both marker types indicate significant differentiation between the Papaloapan population and all remaining localities. In the mtDNA study [16], three haplotypes (4E, 5E, and 7E) that are part of the divergent mitochondrial “PAP” lineage were found in Papaloapan. The analysis of microsatellite variation showed that Papaloapan had the highest number of private alleles and F_ST_ tests confirmed that this population is divergent from the other populations. The STRUCTURE analysis revealed that all individuals from Papaloapan were homogeneously grouped into one cluster for K = 3 (Fig. 2, Fig. 3), and at higher values of K (up to K = 12), Papaloapan individuals remained in one homogeneous cluster, and distinct from all other studied individuals.

Although both mtDNA and nuclear microsatellites thus reveal unique signatures in the population from Papaloapan, the marker classes yield somewhat conflicting signals about the gene flow patterns across the biogeographic break that separates this population from the rest of the range of *D. mawii*. For mtDNA, distinct haplotypes are found at relatively low frequency in Papaloapan, while additional haplotypes are shared with adjacent populations to the east of the isthmus. This indicates mitochondrial gene flow westwards across the isthmus, into Papaloapan [16]. Nuclear microsatellites appear to indicate trans-isthmian gene flow in the opposite direction. Assuming that the STRUCTURE signals for K = 3 indicate an ancient evolutionary separation of a population to the west of the isthmus, occurrence of that cluster today in regions to the east of the isthmus (Fig. 3) would suggest recent eastward gene flow. Differential gene flow signals from mtDNA and nuclear microsatellites could be revealing different episodes of trans-isthmian gene flow (potentially reflecting Pleistocene climatic oscillations leading to temporary links between now-distinct drainages), reflect the stochastic nature of lineage coalescence, or be due to a sex bias in dispersal (leading to different patterns for the maternally inherited mtDNA compared to biparentally inherited nuclear loci).

Another alternative is to interpret the “black” cluster from Fig. 2 and Fig. 3 as a signal of long-term frequent gene flow across the isthmus, but lack of admixture with adjacent habitats further to the east, towards the Yucatan. While the latter scenario appears less likely, our data cannot conclusively distinguish between these alternatives.

The uniqueness of the Papaloapan population could be explained by the fact that this is the only population located northwest of the Isthmus of Tehuantepec and the Sierra de Santa Marta (Fig. 1). Several studies have identified this transitional zone (Fig. 1) as an important biogeographic divide [8], [9], [10], [11], [12], [13], [60], where major changes in the distributional patterns occur for many taxa [11]. This barrier separates aquatic species in the Papaloapan basin from those of the other basins [61]. For *D. mawii* we would anticipate genetic structure similar to that found in other vertebrates limited to wetlands. For example, populations of the ornate shrew (*Sorex ornatus*) of southern California exhibit population structure between the unconnected wetlands but not inside each wetland [62]. In the lowland habitats in Central America the red-eyed tree frog (*A*. *callidryas*) shows pronounced genetic structure across the Cordilleran Mountains [14]. Within the geographic range of *D. mawii*, the genus *Bufo* has different genetic lineages to the West and East of the Isthmus of Tehuantepec and Sierra de Santa Marta [12]. This area is geographically very complex, where major tectonic events, such as sea level changes and continental uplift, have created small isolated basins within a very large plain. Sea level oscillations during the Miocene to Pleistocene allowed intermittent connection between the hydrological basins within the State of Veracruz [8], [63], [64], [65], [66]. The hydrological isolation of the Papaloapan basin coincides with the divergence time estimated with mtDNA for *D. mawii* [16].

### Gene flow on the Yucatan peninsula and within the Grijalva-Usumacinta hydrological basin

*Dermatemys mawii* populations on the Yucatan Peninsula are only weakly differentiated from each other for mtDNA, and two haplotypes that differ by only a single mutation dominate this entire region [16]. Our microsatellite data are mostly congruent with this interpretation. Bayesian clustering results (Fig. 2) show one prevailing cluster in this area, many pairwise differentiation estimates from the Yucatan are low and/or non-significant (Tab. 2), and mis-assignment is common among Yucatan populations (Tab. 3). Exceptions from this are the populations from Sacnab and Salpeten, which may be affected by recent losses of genetic variability.

Most of the populations surveyed in this study are located to the southeast of the Isthmus of Tehuantepec in the Grijalva-Usumacinta river basin. *Dermatemys mawii* is highly aquatic and the rivers in this basin have been interconnected for thousands of years. The relatively high levels of gene flow between localities that we observed are, therefore, not unexpected. However, it is notable to find such high levels of gene flow between localities that are separated by long geographic distances (more than 300 km), as suggested by the mis-assignment of individuals to populations that are located at considerable distance from their collection site. Our results also support human-mediated gene flow due to the translocation of turtles to distant areas as an important factor that may have influenced the low level of contemporary genetic structuring.

*D. mawii* has been of great economic importance as food, and it has probably been used for trade or as part of peace offerings to the dominant ruling culture since the time of the Olmecs [16], the first pre-Columbian civilization in Mexico *circa* 3000 years ago [67], [68], [69], [70], [71]. Freshwater turtles have been an important source of animal protein for the Mesoamerican cultures; locals have captured and raised these animals in backyard ponds, until the present times [1]. Turtle remains have been present in all of the important archeological burial sites. As part of the offerings given to important personages, the species could have been a part of the local biota or originated far away from the site. Remains of *D. mawii* were found in the Teotihuacan archeological site more than 200 kilometers from the natural range of distribution of this species [72] and in the Cuicuilco archeological site in the Basin of Mexico more than 300 kilometers from its natural range [72]. These finds are tentative evidence that turtles were translocated from one area to another for trade, tribute or ritual purposes.

One example of a species of turtle that was apparently not strongly affected by recent human translocations is the alligator snapping turtle *Macrochelys temminckii*. This turtle also has a high commercial value, and is distributed in different drainages of the Gulf of Mexico in the USA, but shows very low levels of gene flow for both microsatellites and mtDNA markers [73]. In our analysis, *D. mawii* individuals were frequently assigned to localities far away from their collection sites. Although assignment quality might improve with a larger number of loci, far-reaching gene flow in *D. mawii* could be explained by the long distance connection of rivers within the Grijalva-Usumacinta basin. Alternatively, far-reaching gene flow could be the result of human translocation of individuals, as further supported by mtDNA data [16]. Due to the high economic value of the species, this process may have been going on for hundreds or even thousands of years [16]. A similar situation was revealed for the diamondback terrapin (*Malaclemys terrapin*), where mixing of lineages was found to be due to animal translocation during the early twentieth century [74]. Another example of human translocation of turtles is the Radiated Tortoise (*Geochelone radiata*) [75], where releases of captive animals by poachers have strongly shaped contemporary population structuring. Gopher tortoises (*Gopherus polyphemus*), which have been moved by humans, show high levels of incorrect assignment to their collection localities [28].

### Cryptic population structuring in the southern and south-eastern regions

For individuals with the divergent mtDNA haplotype 1D (up to 2% divergent from all remaining haplotypes [14], a genetic distance indicative of species-level distinction in some turtle lineages [16]), it was not straightforward to interpret the results based on nuclear loci. While those individuals appeared clearly divergent in the FCA analysis (Fig. 4), the STRUCTURE analyses (Fig. 2, Fig. 3) did not separate ‘1D’ individuals from other conspecifics. We suggest that this apparent incongruence among analysis methods could be due to sampling bias associated with the low number of individuals carrying the 1D haplotype (n = 4) included in our study. STRUCTURE uses individual-level information on allele frequencies to generate clusters that are in Hardy-Weinberg and linkage disequilibrium. For this, reasonable sample sizes are necessary to accurately estimate population-specific allele frequencies. FCA on the other hand analyses one individual at a time, only using presence/absence information for individual alleles. This likely allows for informative individual-based analyses, even when only few individuals from a divergent deme are included in the data set.

Consistent with this view, the four individuals carrying the mitochondrial 1D haplotype had, despite their low sample size, an abundance of private alleles when compared to all remaining individuals. HP-RARE estimated their private allelic richness to 0.8, compared to 2.7 in all 249 remaining samples. Without applying such a correction for sample size, the four 1D individuals combined had 3 private alleles, and at four additional alleles they showed frequency differences larger than 0.35 compared to those allele frequencies in the remaining individuals. In summary, mtDNA and microsatellites both tentatively indicate cryptic evolutionary distinctiveness of some individuals in the southern and south-eastern end of the range of *D. mawii*. Additional analyses of individuals in this area will be necessary to assess the taxonomic relevance of the mitochondrial 1D lineage.

### Conservation implications

Based on the slightly reduced levels of genetic variability in some populations, especially in Sacnab and Salpeten, we recommend that additional field surveys be conducted in these areas to assess their current conservation status, and to ensure their future survival. Lower genetic variability of some populations on the Yucatan Peninsula could be explained by recent genetic drift, suggesting that sustained gene flow in such homogeneous habitats may be important for the long-term genetic health of these populations.

Genetics can inform conservation planning and management by identifying evolutionary distinct lineages and local population structuring. In a previous study of mtDNA in *D. mawii* [16], recognition of one evolutionary significant unit for the localities populated by haplotype 1D, and one MU for populations in the rest of the localities was recommended. A reevaluation of those recommendations now needs to be undertaken to incorporate the results of this study into conservation/management plans. We recommend managing *D. mawii* as a single broad MU because of the pattern of genetic admixture that we observed throughout the range of this species [76], [77], [78]. However, because we found specific signatures of genetic differentiation between Papaloapan and the other populations for both mitochondrial and nuclear markers, we recommend that the Papaloapan population be given special priority conservation protection under the auspices of the laws of the Mexican Fish and Wildlife Service (Article 61b), which emphasizes the importance of the maintenance of genetically diverse populations [79]. Similarly, we suggest that the Salinas and Sarstun populations also be given special priority conservation protection, until a more in depth study can be completed to elucidate the taxonomic affinities and evolutionary significance of *D. mawii* individuals carrying the divergent 1D haplotype found in the Salinas and Sarstun populations.

We also recommend further studies examining the Salinas and Sarstun populations (containing individuals with the divergent mitochondrial 1D lineage) to investigate if some level of assortative mating among individuals belonging to different mitochondrial lineages may exist. In addition, implementation of morphometric analyses of individuals from these populations in order to determine if there are patterns of morphological differentiation correlated with genetic differentiation [80] seems highly warranted. Finally, we recommend performing field studies in these areas in order to better document and elucidate any ecological or behavioral differences between individuals from different genetic groups.

## Supporting Information

Figure S1 **Plot of isolation by distance for** ***D. mawii*** **populations, with genetic distance plotted against geographic distance** [**42**] **.**
(TIF)

Figure S2 **Evaluation of STRUCTURE** [**44**] **results: plot of Evanno et al.**'**s ΔK** [**46**] **for different numbers of clusters (values of K).**
(TIF)

Figure S3 **Evaluation of STRUCTURE** [**44**] **results: plot of lnP(D) for different numbers of clusters (values of K).**
(TIF)
