# Supplementary figures and images for: Cryptic Population Structuring and the Role of the Isthmus of Tehuantepec as a Gene Flow Barrier in the Critically Endangered Central American River Turtle
Source: PLoS One. 2013 Sep 25;8(9):e71668. doi: 10.1371/journal.pone.0071668 (PMC3783458; doi:10.1371/journal.pone.0071668)

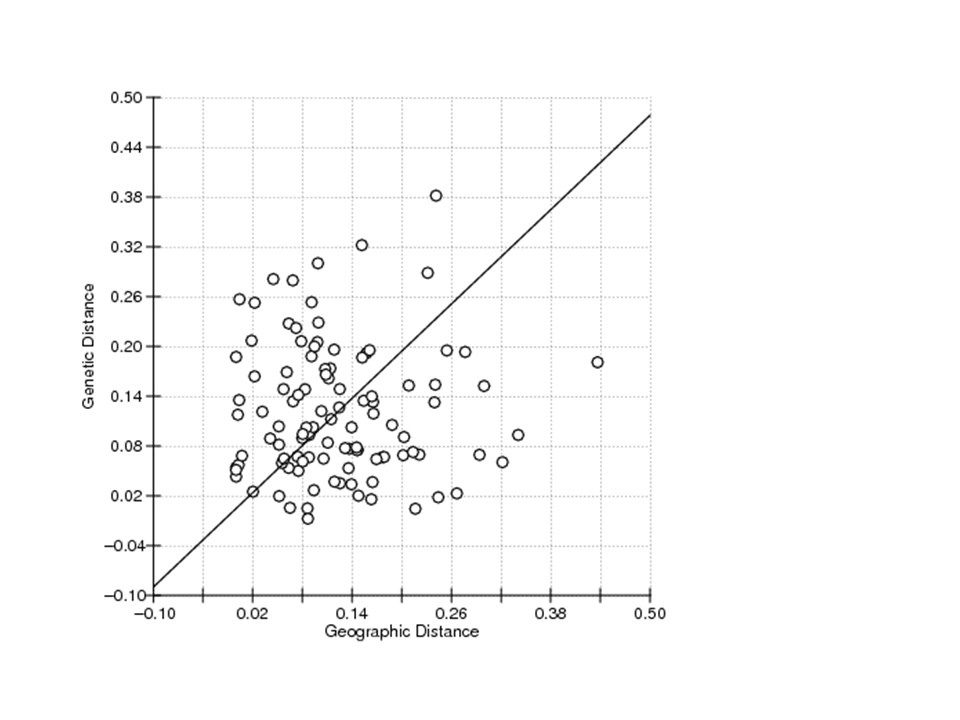

Supplement: Figure S1 — Plot of isolation by distance for D. mawii populations, with genetic distance plotted against geographic distance [42] . (TIF) [file pone.0071668.s001.tif]
